# Differences between manual CPR and corpuls cpr in regard to quality and outcome: study protocol of the comparing observational multi‐center prospective registry study on resuscitation (COMPRESS)

**DOI:** 10.1186/s13049-021-00855-9

**Published:** 2021-02-25

**Authors:** S. Seewald, S. Dopfer, J. Wnent, B. Jakisch, M. Heller, R. Lefering, JT Gräsner

**Affiliations:** 1 Institute for Emergency Medicine and Department of Anesthesiology and Intensive Care Medicine, University Hospital Schleswig-Holstein, Campus Kiel, Arnold-Heller-Straße 3, 24105 Kiel, Germany; 2 Elektromedizinische Geräte G. Stemple GmbH, Kaufering, Germany; 3 School of Medicine, University of Namibia, Windhoek, Namibia; 4 Kreis Ploen, Preetz, Germany; 5 Institute for Research in Operative Medicine, Faculty of Health, University of Witten/ Herdecke, Witten, Germany

**Keywords:** Corpuls cpr, GS Elektromedizinische Geräte G. Stemple GmbH, Mechanical CPR, German resuscitation registry

## Abstract

**Background:**

The effect of mechanical CPR is diversely described in the literature. Different mechanical CPR devices are available. The corpuls cpr is a new generation of piston-driven devices and was launched in 2015. The COMPRESS-trial analyzes quality of chest compression and CPR-related injuries in cases of mechanical CPR by the corpuls cpr and manual CPR.

**Methods:**

This article describes the design and study protocol of the COMPRESS-trial. This observational multi-center study includes all patients who suffered an out-of-hospital cardiac arrest (OHCA) where CPR is attempted in four German emergency medical systems (EMS) between January 2020 and December 2022. EMS treatment, in-hospital-treatment and outcome are anonymously reported to the German Resuscitation Registry (GRR). This information is linked with data from the defibrillator, the feedback system and the mechanical CPR device for a complete dataset.

Primary endpoint is chest compression quality (complete release, compression rate, compression depth, chest compression fraction, CPR-related injuries). Secondary endpoint is survival (return of spontaneous circulation (ROSC), admission to hospital and survival to hospital discharge). The trial is sponsored by GS Elektromedizinische Geräte G. Stemple GmbH.

**Discussion:**

This observational multi-center study will contribute to the evaluation of mechanical chest compression devices and to the efficacy and safety of the corpuls cpr.

**Trial registration:**

DRKS, DRKS-ID DRKS00020819. Registered 31 July 2020.

## Background

Chest compression is the key procedure during cardiopulmonary resuscitation. High quality chest compression is essential for survival with favorable neurological recovery after cardiac arrest [1]. With manual CPR, increasing fatigue of rescuers and frequent interruptions of compressions have been reported [2]. Both fatigue and interruptions decrease chest compression quality and the generated blood flow. High performance chest compressions have been shown to improve survival rates, compared to insufficiently performed chest compressions [3, 4].

For that reason, several mechanical CPR devices with different compression methods have been developed. These devices perform chest compressions through inflatable vests, mechanical pistons or load distributing bands. The most frequently used devices are LUCAS (piston-driven) (manufactured by Stryker Medical) and AutoPulse (load distributing band) (manufactured by ZOLL medical).

Based on the premise that mechanical CPR provides a sustained and improved quality of chest compressions, an optimized outcome might be expected through mechanical chest compression devices [5]. Nevertheless, studies investigating the effect of mechanical CPR show differing results. Meanwhile, three large, high-quality, prospective, multi-center randomized controlled trials on mechanical CPR have been published, as well as several publications from retrospective registry analyses [6–9]. Findings were inconsistent and contradictory, leaving it uncertain if mechanical chest compressions during CPR may improve the outcome of patients suffering cardiac arrest. Also, several systematic reviews were unable to show a benefit for mechanical CPR [10–12].

Therefore, the European Resuscitation Council (ERC) Guidelines do not recommended the use of mechanical chest compression devices routinely. However, these devices are a reasonable alternative in situations where sustained high-quality manual chest compressions are impractical or compromise provider safety (e.g. during transport or coronary angiography). The current recommended indications for mechanical CPR in Germany (following the international recommendations) are: [13].

- ongoing resuscitation and limited personal resources.
- transport with ongoing CPR if indicated..

If mechanical CPR devices are used, interruptions of chest compressions due to device deployment should be avoided [14].

**Fig. 1:**
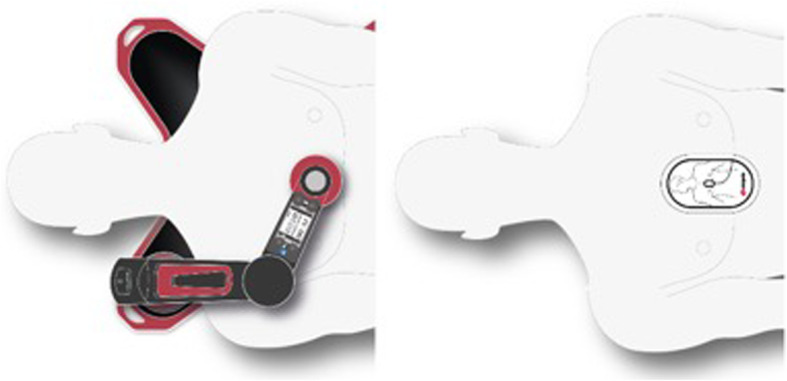
Illustration of the setup; Left: Mechanical CPR with corpuls cpr (CPR performance recorded by corpuls cpr); Right: Manual CPR with corPatch CPR feedback sensor (recorded by corpuls3). © GS Elektromedizinische Geräte G. Stemple GmbH

The corpuls cpr is a new generation of piston-driven device, manufactured by GS Elektromedizinische Geräte G. Stemple GmbH. It allows increased flexibility due to a rotatable arm (Fig. 1) and enables therapy based on the following settings:

- Compression rate: 80 to 120 compressions per minute (adjustable in increments of 1 compression per minute).
- Compression depth: 2–6 cm (adjustable in increments of 0.1 cm).
- Therapy mode: 30:2 / 15:2 / continuous.
- Patient’s chest height: 14 to 34 cm.
- Patient’s weight: no limitation.
- Operating time per battery: 90 minutes (typically).

The rollout of the corpuls cpr took place in 2015.

The corpuls cpr has not been studied in humans before. However, the performance between the new device and an existing piston-driven mechanical CPR device varies: Eichhorn et al. found a higher mean arterial pressure during resuscitation in an animal model when using the corpuls cpr [15]. Therefore, this observational multi-center study will evaluate the efficacy and safety of the corpuls cpr in humans.

## Methods and design of the COMPRESS-trial

The aim of the COMPRESS-trial is to analyze high performance CPR by manual CPR and the corpuls cpr and to evaluate the feasibility and safety of the device.

### Participating EMS

The German EMS is a two-tiered, paramedic and emergency physician-based system. Paramedic-staffed ambulances (mobile advanced life support (ALS) units) are dispatched for basic aid and patient transportation. If necessary, like in a cardiac arrest, medic vehicles carry emergency physicians to the incident location. The physicians are mainly anesthetists, surgeons, and internists after completing additional training in emergency medicine.

Four German Emergency Medical Services (EMS) participate in the COMPRESS-trial: Aachen (255,967 inhabitants), Dortmund (601,000 inhabitants), Göppingen (254,618 inhabitants) and Gütersloh (371,429 inhabitants). These centers fulfill the following requirements: use of the defibrillator corpuls 3 exclusively (manufactured by GS Elektromedizinische Geräte G. Stemple GmbH); previous experience in the use of the corpuls cpr; the corpuls cpr is available at the physician staffed unit; participation in the German Resuscitation Registry including collection of in-hospital outcome data; they are not participating in a clinical study that would be in conflict with the COMPRESS-trial. Details about the covered population in the German Resuscitation Registry are shown in the public annual report [16]. Demographic data of the four participating EMS are presented in Table 1. The percentage of mechanical CPR in Germany is around 20 %.

**Table 1:** Demographic and process data of the four EMS participating in the COMPRESS-trial. Hours on duty: Sum of hours on duty of all physician staffed EMS units in that region per year

Site	Population covered	Area covered (km^2^)	Population density (inhabitants/km^2^)	Hours on duty of the physician staffed unit (h)	CPR incidence (1/100,000 inhabitants and year)	estimated number of attempted CPR per year
Aachen	255,967	162	1,580	17,520	52.7	135
Dortmund	601,000	280	2,146	52,560	65.9	396
Göppingen	254,618	642	397	29,930	67.2	171
Gütersloh	371,429	969	383	38,532	54.4	202

### German Resuscitation Registry

Data collection and analysis are performed based on the German Resuscitation Registry database. The registry is operated by the German Society of Anaesthesiology and Intensive Care Medicine and was founded in 2007. The German Resuscitation Registry is financed by the participants and the German Society of Anaesthesiology and Intensive Care Medicine. Participation in the registry is voluntary. The participating centres pay an annual fee. The registry includes fully anonymized data from out-of-hospital cardiac arrest (OHCA), in-hospital cardiac arrest (IHCA), and in-hospital emergency treatment. Multiple plausibility checks have been implemented into this application in order to assure data quality [17, 18].

The German Resuscitation Registry for OHCA is divided into two different datasets:

1. The ‘Pre-hospital’ dataset is focussed on documentation of pre-hospital logistic issues, presumed aetiology, resuscitation treatment, and patients’ initial outcomes and includes 118 variables [19, 20].
2. The **‘**Post resuscitation care’ dataset is aimed at documentation of in-hospital post-resuscitation efforts. Participating hospitals can choose a basic version and an extended version with 156 variables [19].

Within the COMPRESS-trial additional data is necessary and recorded in a special dataset in the German Resuscitation Registry:

The ‘COMPRESS-trial’ dataset allows the documentation of resuscitation related injuries, the method of examination and the first blood gas analysis after hospital admission. The included variables are shown in Table 2.

**Table 2:** Variables of ‘COMPRESS-trial’ dataset. CT = computer tomography; pO2 = oxygen partial pressure; pCO2 = carbon dioxide partial pressure; Hb = hemoglobin

variable	specification
rib fracture	yes no
serial rib fracture	yes no
sternal fracture	yes no
pneumothorax	yes no
hematopneumothorax	yes no
intra-thoracic bleeding	yes no
intra-abdominal bleeding	yes no
How was the injury diagnosed?	routine diagnostic suspicion based diagnostic
Which diagnostics were performed?	CT x-ray ultrasound
pO2 in first blood gas analysis after hospital admission	
pCO2 in first blood gas analysis after hospital admission	
Hb in first blood gas analysis after hospital admission	

### Study enrollment and data management

The observational non-interventional COMPRESS-trial includes all patients with OHCA and resuscitation attempted between January 2020 and December 2022 in the four participating EMS. The emergency physician on scene decides whether manual or mechanical CPR is used. The choice is not influenced by this trial. To avoid selection bias based on the COMPRESS-trial, there is no modification of the local treatment strategy. If manual CPR is used, a feedback system is recommended.

The EMS treatment is anonymously recorded in the ‘pre-hospital’ dataset of the German Resuscitation Registry. If the patient is admitted to hospital the EMS or the hospital collects the ‘post-resuscitation-care’ dataset in at least the basic version as well as the ‘COMPRESS-trial’ dataset.

Inclusion criteria:

- OHCA and resuscitation attempted between January 2020 and December 2022.
- Patient-age: 8 years or older (according to the technical approval of the corpuls cpr)..

Exclusion criteria:

- Traumatic cause of cardiac arrest.
- Missing core data (e.g. data of corpuls cpr, survival data)..

The quality of manual chest compression is measured by a feedback system in the defibrillator. A real-time feedback system incorporated into the corpuls3 defibrillator (with corPatch CPR feedback sensor) is used in this study. The feedback sensor is placed on the patient’s sternum during resuscitation. The feedback system issues auditory and visual prompts if deviations from the guideline recommendations are detected [21]. The quality of mechanical chest compression is recorded by the corpuls cpr.

The data files of the corpuls3 (defibrillator) and, if used, of the corpuls cpr are collected on a local server in the EMS. These data files are anonymized and matched with the German Resuscitation Registry dataset.

Interim and the final analysis of chest compression quality is analyzed by a manual annotation tool combined with script-based data processing (provided by GS Elektromedizinische Geräte G. Stemple GmbH). Then the time of resuscitation is divided into “manual chest compression” and “mechanical chest compression”. Missing data is excluded. In each group the percentage of correct chest compressions are recorded. The correct depth based on the ERC Guidelines is defined as a minimum of 5 and maximum of 6 cm of depth and the correct release having no more than 25 N remaining on the chest. The overall time of chest compression is divided into “compression-time” and “no-compression-time” (e.g. ventilation, defibrillation). In the compression time, the compression rate is calculated. The chest compression fraction (compression-time / no-compression-time) is calculated for the entire resuscitation in both groups.

### Training

Before the study started, paramedics and physicians in each participating EMS were able to join a training in manual CPR and the use and deployment of the corpuls cpr. The training was supervised by the sponsor and was performed by the Institute for Emergency Medicine, University Hospital Schleswig-Holstein.

### Patient consent

The COMPRESS-trial is an observational non-interventional study with a non-inferiority design. Patient treatment is based on international guidelines and local protocols and not influenced by the trial.

The recorded and analyzed data are completely anonymous and are routinely recorded for quality management purpose. Therefore, patient consent is not required.

The study was approved by the ethics committee of the University of Kiel (Ref. no.: D 422/19) and the scientific advisory board of the German Resuscitation Registry (Ref. no.: 2020_03).

### Analysis

This study analyzes the influence and safety of mechanical CPR in daily practice in Germany. Therefore, the COMPRESS-trial is an observational non-interventional study analyzing comparing corpuls cpr with manual CPR.

The primary endpoint of the study is quality of chest compression:

- Ratio of complete release: How often is full recoil by total release between two compressions ensured (no more than 25 N remaining on the chest)?
- Compression rate: How often is a compression rate between 100 and 120/min reached? (Percentage of correct compressions compared to all compressions)
- Compression depth: How often is a compression depth between 5 and 6 cm reached? (Percentage of correct compressions compared to all compressions) How often was the compression too deep (> 6 cm) or shallow (< 5 cm)?
- Chest compression fraction: How was the rate of compressions compared with the duration of resuscitation?
- Resuscitation-related injuries (Table 2)..

The secondary endpoint of the study is outcome:

- ROSC
- Hospital admission with ROSC or ongoing CPR.
- Survival at hospital discharge.
- Influence of chest compression technique on first blood gas analysis at hospital admission..

Annual interim analyses are performed to identify adverse events. If there is an unexpectedly high proportion of adverse events or new findings regarding the corpuls cpr that decrease the benefit/risk ratio, which can’t be fixed by corrective actions, the trial is terminated and the sponsor informs the authority (Bundesinstitut für Arzneimittel und Medizinprodukte, BfArM).

Within the observed groups, patient characteristics and reasons for the use of a mechanical CPR device are analyzed in parametric and non-parametric tests. Chest compression quality (primary endpoint) and outcome (secondary endpoint) are compared in univariate analysis between the two groups. If the patient is admitted to hospital with ongoing resuscitation the airway management and the first blood gas analysis are analyzed.

With a multivariate regression analysis, the influence of the corpuls cpr on survival is analyzed by adjustment for confounding variables collected in the German Resuscitation Registry based on the existing studies (e.g. age, sex, initial ECG, etiology of cardiac arrest, no-flow-time, low-flow-time,…) [17, 18].

### Role of funding source

GS Elektromedizinische Geräte G. Stemple GmbH funded the training, the COMPRESS-trial dataset in the German Resuscitation Registry and the study management in the Institute of Emergency Medicine at the University Hospital Schleswig-Holstein. One person from GS Elektromedizinische Geräte G. Stemple GmbH is a nonvoting member of the steering committee of the study. GS Elektromedizinische Geräte G. Stemple GmbH had a minor role in the design and conduct of the study; collection, management, analysis and interpretation of the data; preparation or approval of the manuscript. The role of the sponsor and the data management are observed by the notifed body (TÜV Süd). Before submission of the study protocol the sponsor was allowed to review and comment on the manuscript. The investigators are under no obligation to incorporate any such input. The decision to submit for publication will be by the authors and not by the sponsor.

The EMS organization and the EMS personal on scene receive no payment or reimbursement.

## Discussion

The COMPRESS-trial is an observational multi-center study. There are several differences compared to previous randomized trials on mechanical CPR devices. Within this observational study there are no modifications on standard treatments in the participating EMS. Therefore, the paramedics and emergency physicians are minimally affected by the fact that they are part of a trial and patients can receive a treatment according to current guidelines. As a result, the device is only applied when indicated. Therefore, the resulting structural differences are adjusted in a multivariate analysis to reach a comparison between the manual and mechanical chest compression group.

Besides the differences resulting from the design of the trial, the COMPRESS-trial investigates aspects that lack evidence:

For instance, effective airway and ventilation strategies when a chest compression device is applied are missing [22]. Therefore, the dataset includes information on airway device, capnography and measurements for pCO2, pO2 and Hb from the first blood gas analysis after hospital admission.

It is unclear why previous studies found controversial results for mechanical CPR. The outcome between the devices differs perhaps due to the different chest compression technologies. That is why more evidence for mechanical chest compression devices is needed.

## Conclusions

The COMPRESS-trial will contribute to the evaluation of mechanical chest compression and to the efficacy and safety of the corpuls cpr.

## Data Availability

Not applicable.

## Abbreviations

ALS: Advanced life support
COMPRESS: Comparing Observational Multi-center Prospective Registry Study on Resuscitation
CPR: Cardiopulmonary resuscitation
CT: Computer tomography
ECG: Electrocardiogram
Hb: Hemoglobin
IHCA: In-hospital cardiac arrest
OHCA: Out-of-hospital cardiac arrest
pO2: Oxygen partial pressure
pCO2: Carbon dioxide partial pressure
ROSC: Return of spontaneous circulation
